# Applying evolutionary concepts to wildlife disease ecology and management

**DOI:** 10.1111/eva.12168

**Published:** 2014-05-31

**Authors:** Eric Vander Wal, Dany Garant, Sophie Calmé, Colin A Chapman, Marco Festa-Bianchet, Virginie Millien, Sébastien Rioux-Paquette, Fanie Pelletier

**Affiliations:** 1 Département de biologie, Université de SherbrookeSherbrooke, QC, Canada; 2 El Colegio de la Frontera SurChetumal, Quintana Roo, Mexico; 3 Department of Anthropology and McGill School of Environment, McGill UniversityMontreal, QC, Canada; 4 Wildlife Conservation SocietyBronx, New York, NY, USA; 5 Redpath Museum, McGill UniversityMontreal, QC, Canada

**Keywords:** conservation, eco-evolutionary dynamics, environmental change, epidemiology, host–pathogen interactions, zoonosis

## Abstract

Existing and emerging infectious diseases are among the most pressing global threats to biodiversity, food safety and human health. The complex interplay between host, pathogen and environment creates a challenge for conserving species, communities and ecosystem functions, while mediating the many known ecological and socio-economic negative effects of disease. Despite the clear ecological and evolutionary contexts of host–pathogen dynamics, approaches to managing wildlife disease remain predominantly reactionary, focusing on surveillance and some attempts at eradication. A few exceptional studies have heeded recent calls for better integration of ecological concepts in the study and management of wildlife disease; however, evolutionary concepts remain underused. Applied evolution consists of four principles: evolutionary history, genetic and phenotypic variation, selection and eco-evolutionary dynamics. In this article, we first update a classical framework for understanding wildlife disease to integrate better these principles. Within this framework, we explore the evolutionary implications of environment–disease interactions. Subsequently, we synthesize areas where applied evolution can be employed in wildlife disease management. Finally, we discuss some future directions and challenges. Here, we underscore that despite some evolutionary principles currently playing an important role in our understanding of disease in wild animals, considerable opportunities remain for fostering the practice of evolutionarily enlightened wildlife disease management.

## Introduction

Evolutionary dynamics play an important role in why and how we should manage wildlife disease (Hudson et al. 2002; Karesh et al. 2012). In an increasingly connected world, the threat of spreading existing and emerging pathogens is growing (Daszak et al. 2000) and in some cases devastating (e.g. Jensen et al. 2002; Jancovich et al. 2004; Fenton 2012). When a pathogen is transported across natural barriers by human actions, it can often have significant negative impacts upon naïve hosts for which it may represent an entirely new selective pressure (Daszak et al. 2000) with the potential to cause extinction (De Castro and Bolker 2005). Moreover, growing movements of people and international trade in livestock and food products will inevitably increase the spread of exotic diseases (Olden et al. 2004). Therefore, managing wildlife diseases, particularly those of ecological or socio-economic concern, is an increasing challenge. Significant advances have been made to incorporate ecological principles into the study of infectious disease in wildlife (Tompkins et al. 2011), and increasingly, theory is guiding wildlife disease management (Joseph et al. 2013). However, apart from landscape genetics (Real and Biek 2007), the application of evolutionarily enlightened management (Ashley et al. 2003) to wildlife disease remains underexploited (Vander Wal et al. 2014).

The lack of integration of evolutionary principles is surprising given that infectious disease dynamics are an evolutionary interaction between two and more species: host(s) and pathogen(s) (Karesh et al. 2012). Hosts evolve to reduce the costs of infection in three ways: changing behaviours (e.g. avoidance), resistance (i.e. limiting the pathogen burden) or tolerance (i.e. limiting the damage performed by the pathogen burden, Medzhitov et al. 2012). Each of these tactics has different evolutionary implications. For instance, while resistance has a negative effect on the pathogen-creating selective pressure, tolerance does not (Raberg et al. 2009). Where pathogens are exposed to selection, however, they must evolve to continue to exploit their hosts (Hudson et al. 2002). For the pathogen, this ensures that the basic reproductive rate (*R*_0_) remains >1; that is, prior to a host’s death, it will infect at least one new susceptible individual. As such, even among the most virulent pathogens, evolution of reduced virulence (Boots and Mealor 2007) is one of the hallmarks of pathogen evolution which follows the infection of a naïve host population. Some emerging pathogens now coexist within their host [e.g. myxoma virus in European rabbits, *Oryctolagus cuniculus* (Fenner 2010), chydrid fungus and amphibians (Phillips and Puschendorf 2013)]. However, when hosts fail to adapt rapidly enough to novel pathogens or pathogens fail to evolve lower virulence, the threat of host extinction remains [e.g. devil facial tumour disease (McCallum 2008), white-nose syndrome (Blehert et al. 2009), Fig. 1]. Predominantly, our evolutionary lens has been focused on pathogen evolution – typically thought to occur on shorter timescales than host evolution (Grenfell et al. 2004). We argue, however, that in addition to historical timescales, mounting evidence for rapid evolution (Hairston et al. 2005), suggests that evolutionary principles provide insights on the management of host, pathogen and host–pathogen dynamics. These insights, including inferences into the origins of emergent diseases, into rates of local or landscape-scale disease spread, or into pathogens or environments as selective agents and their downstream effects on population dynamics as a function of changing host-life history.

**Figure 1:**
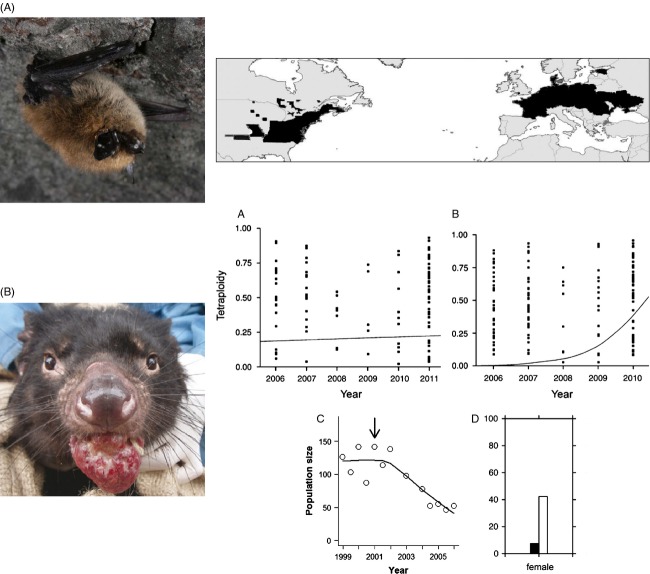
Two examples of critical wildlife diseases for which evolutionary concepts [e.g. phylogenetics (A, Chaturvedi et al. 2010) or selection (B, Ujvari et al. 2014)] are important in understanding host-pathogen dynamics. (A) White-nose syndrome has caused unprecedented declines in bat populations in North America (Fenton 2012). The causal agent of white-nose syndrome is a fungus, *Geomyces destructans* (Gd). The fungus is cold-adapted and infects bats largely in their hibernacula where it disrupts patterns of torpor resulting in increased mortality due to desiccation (Warnecke et al. 2012). An emergent disease in North American bats, Gd is thought to be of European origins where it does not cause increased mortality (Blehert 2012). Below is a distribution of Gd [modified from Puechmaille et al. (2011) to approximate information from www.whitenosesyndrome.org, accessed 25-02-2014]. Differences in pathogenesis on North American and European bats is not well understood (Cryan et al. 2013). However, these may be ecological, for example, population dynamics or overwintering environment, or alternately they may be a function of evolved differences in host species (Blehert 2012) or due to lateral gene transfer of species recombination (Puechmaille et al. 2011). Photo Credit (Blehert 2012). (B) Devil facial tumour disease (DFTD) is a cancer horizontally transmitted by biting among devils (*Sarcopilus harrisii*) (Hamede et al. 2012). First discovered in 1996, it has had devastating effects on devil populations. Cancers are novel areas for the application of evolution to the biology of disease (Nesse and Stearns 2008). Recent evidence suggests that devil removal programmes are correlated with accelerated evolution of tetraploidy. Tetraploidy is, in turn, thought to favour slower tumour growth (Ujvari et al. 2014). Compare, for example, (a) proportion of tetraploidy through time for 10 populations with no disease suppression trials versus (b) a population subject to disease suppression (modified from Ujvari et al. 2014). Additionally, the spread of disease has caused a change in life-history traits (Jones et al. 2008b; Lachish et al. 2009); (c) illustrates the population decline following the emergence (arrow) of DFTD in one population (Freycinet, data adapted from McCallum et al. 2007). (d) Illustrates the concurrent change in primiparity pre- (black bars) and post (white bars) invasion of DTFD in that same population of devils (reproduced from Jones et al. 2008a). Photo Credit (McCallum 2008).

**Figure 2:**
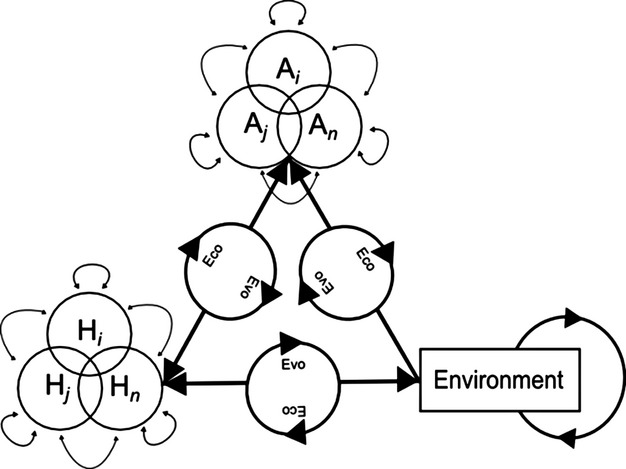
A conceptual update of the Epidemiological Triangle to include evolutionary ecology, where *H*_*i*_ through *H*_*n*_ are the host community and *A*_*i*_ though *A*_*n*_ are the infectious pathogens.

In many practical instances, the management of wildlife diseases has involved collaboration between clinical veterinarians, veterinary epidemiologists, and at times, wildlife managers. Yet our understanding of these diseases has largely been shaped by evolutionary ecologists such as May and Anderson (1983). This distinction reinforces separations outlined in Tinbergen’s Four Questions (Tinbergen 1963; Nesse and Stearns 2008). The former group of professionals focuses on proximate mechanisms of disease (i.e. ‘causation’ and ‘ontogeny’); for example, aetiology or pathogenesis. The latter concentrate instead on the ultimate or evolutionary causes of disease (i.e. ‘survival value’ and ‘evolution’ or phylogeny). To understand the ultimate causes of disease spread, we must answer such questions as how pathogens can increase *R*_0_ or how hosts adapt to emergent diseases. Where proximal methods are important for diagnosing and treating individuals, the primary focus of wildlife managers is population level indices of ‘health’, such as population growth, which can be affected by disease. The need remains for a more comprehensive synthesis of our understanding of wildlife disease from individual hosts to ecosystems (Tompkins et al. 2011). For instance, aspects of disease linking different parts of an ecosystem include pathogen transmission that can vary within individual hosts due to heterogeneity in contact rates or immunity; transmission among multiple hosts and involves multiple pathogens; and occurs in environments with successional trajectories that affect which hosts reside within them (Fig. 2). Ultimately, evolutionary principles can inform management strategies (Ashley et al. 2003; Hendry et al. 2011) and should help predict how species may or may not adapt when facing the selective pressures imposed by novel infectious pathogens, changing environments or management interventions.

In this review, we first introduce the eco-evo epidemilogical triangle, and update of the epidemiological that can then be seen as a rubric to include evolutionarily enlightened principles into the study and management of wildlife disease. Within this framework, we then explore the evolutionary implications of environment–disease interactions in the light of climate change and rapid anthropogenic changes to landscapes. Next, we synthesize areas where applied evolution can be employed in wildlife disease management. Finally, we discuss some future directions and challenges that exist for evolutionarily enlightened wildlife disease management.

## The eco-evo epidemiological triangle

A typical pedagogical framework for understanding wildlife disease ecology begins with the ‘epidemiological triangle’(Wobeser 2006; Scholthof 2007). First introduced in the 1960s (McNew 1960), the epidemiological triangle suggests that three components are necessary for persistence of infectious disease: host, pathogen and environment (for vectorborne diseases this is necessarily more complex; see Wobeser 2006). Albeit unrealistic, this basic framework begins as static. All three components, however, are involved in complex community interactions, including competition among multiple hosts (*H*_*i*_) or among multiple pathogens (*A*_*i*_) relying on the same host (Hudson et al. 2002; Fig. 2). These interactions are also affected by natural or anthropogenic environmental change (Wilcox and Gubler 2005), potentially leading to co- or eco-evolutionary dynamics (Duffy and Forde 2009).

Eco-evolutionary dynamics describe a feedback (or correlation) between evolutionary responses and ecological processes (Schoener 2011). Traditionally, evolutionary ecology was the study of how ecological processes shaped evolutionary responses. However, it is now known that evolutionary responses can also shape ecological processes (Smallegange and Coulson 2013). For instance, changing distribution of a trait in a population (e.g. the evolution of body size) can affect ecological processes such population growth rates (Pelletier et al. 2007). Co-evolution is a special case of eco-evolutionary dynamics involving two species (Pelletier et al. 2009). Co-evolution describes reciprocal adaptive genetic changes between two species. For example, co-evolution occurs when a pathogen acts as a selective agent differentially affecting the survival and fecundity of its host. In turn, the host evolves strategies to minimize the costs of infection (e.g. behavioural, resistance, tolerance). Consequently, the pathogen may have to adapt to ensure *R*_0_ remains >0.

European rabbits in Australia are a classic example of the evolutionary interplay between host and pathogen (May and Anderson 1983) involved in the epidemiological triangle. Co-evolution occurred postexposure to novel pathogens [myxoma virus (genera *Leporipovirus*), and rabbit haemorrhagic disease (RHD)] introduced to control the rabbits (Saunders et al. 2010). Release of the myxoma virus in the 1950s was the first attempt at biological population control. Following the initial population collapse, there was rapid natural selection in the pathogen for reduced virulence (Best and Kerr 2000). In 1970s, populations were recovering, and rabbit fleas were introduced to improve the efficacy of myxoma virus transmission. In 1990s, populations were recovering and RHD was released, possibly selecting for earlier age of primiparity (Cooke 2002; Mutze et al. 2002), a host-life history adaptation to compensate for lower survival rates. This life-history response to myxoma led to feedback on the host population dynamics.

Another example of host–pathogen adaptation is facial tumour disease (DFTD, Fig. 1) in devils (*Sarcopilus harrisii*). First discovered in 1996, DFTD is an infectious cancer spread among devils via biting (Hamede et al. 2012). The transmissible cancer cells are genetically different from host cells (McCallum 2008). DFTD is fatal, with mortality occurring at or before age two, prior to the normal age of primiparity (Jones et al. 2008b). It has resulted in dramatic population declines >60% (90% locally) with cascading effect on local ecology (Hollings et al. 2013). DFTD highlights an evolutionary link between host and pathogen in the epidemiological triangle. Firstly, devils in populations exposed to DFTD have been selected to reproduce at an earlier age (Jones et al. 2008b; Lachish et al. 2009). There appears to be an evolutionary response in the cancer (Murchison et al. 2012), with recent evidence suggesting that methylation and changes in gene expression allow the cancer to adapt to its environment via epigenetic alterations favouring tetraploidy (Ujvari et al. 2012). Furthermore, disease suppression trials, involving removing infected animals, may have increased tetraploidy and favour slower growing tumours in the population, further suggesting that the disease is able to responds rapidly to a change in selective environment (Ujvari et al. 2014).

Evolutionary principles have been considered in the conservation response to DFTD, including selective captive rearing of resistant individuals (McCallum 2008). However, in addition to understanding change in the host–pathogen link of the epidemiological triangle, opportunities exist to test whether transmission could be mitigated by capitalizing on the host–environment link. For example, because transmission is dependent on biting during mating and food competition (Hamede et al. 2012), manipulating the distribution of resources may reduce the behaviour that causes transmission. Evidence from raccoons (*Procyon lotor*) suggests that altered resource distribution affects endoparasite transmission as individuals aggregate near a clumped resource (Gompper and Wright 2005; but see Monello and Gompper 2011).

## Environment–disease interactions and the role of evolution

The host–pathogen evolutionary relationship is fundamentally affected by changes in environmental conditions (Fig. 2). Through phenotypic plasticity, genotypes interact with environments (Nussey et al. 2007) to produce different phenotypes for both hosts and pathogens (Mitchell et al. 2005). As a result, changing habitats can have profound effects on host–pathogen evolutionary dynamics (Altizer et al. 2013; Echaubard et al. 2014).

First, environmental change, such as climate warming, can shift species distributions. Such changing selective pressures have implications for management (Ashley et al. 2003). With climate warming, and given sufficient plasticity and/or evolutionary potential, both reservoir hosts and vector species may spread to new latitudes or to higher elevations, promoting the emergence and establishment of a disease in newly invaded regions (Wilcox and Gubler 2005; Jones et al. 2008a). Several vectorborne infectious diseases have recently expanded their range, tracking their hosts whose distribution is tied to climate (Altizer et al. 2013; Simon et al. 2014). Habitat fragmentation could also be important and result in diverse selective effects. For instance, increased agriculture that results in fragmented landscapes can reduce habitat available for ticks that are vectors of several diseases, such as Lyme borreliosis (Ostfeld et al. 1995). Alternately, such fragmented landscapes can create additional edge-habitats, attracting generalists as shown by Manson et al. (1999) for white-footed mice (*Peromyscus leucopus*), an important host in the transmission of Lyme borreliosis (Kurtenbach et al. 2006; Simon et al. 2014).

It is thus vital to realize that the net selective effects of changing landscapes on a disease are the product of the interactions between the effects of these changes on each of the hosts. Quantifying the relative importance of these selective agents will help to predict the evolutionary consequences of habitat fragmentation on disease prevalence. In the previous example, fragmentation had a negative effect on the vector species, *Ixodes* ticks, through restricted migration and thus increased selective pressures, but also fragmentation had a positive effect on several of its hosts, through relaxed selection pressures linked to increased available habitats. In this case, the net resulting selective effect was positive (through increased host density) and translated into persistence of the borreliosis pathogens (Brownstein et al. 2005).

Human-altered landscapes will also affect host–pathogen evolutionary dynamics by decreasing species diversity. This can occur by changing species’ relative abundance in favour of more generalist and smaller species, and promoting the establishment of new species (Hooper et al. 2012). Changes in species diversity will then affect disease prevalence through one of two mechanisms. (i) The dilution effect, which refers to a weakened disease risk by a given pathogen resulting from an increase in species diversity; or (ii) the amplification effect refers to the inverse, an increased disease risk due to increased diversity (Keesing et al. 2006). Keesing et al. (2006) suggest various pathways through which either the dilution or amplification effects could occur, including (i) encounter rate and transmission probability among hosts, (ii) density of susceptible hosts and (iii) mortality and recovery rates of infected hosts. While some studies provide support for a role of host species richness in changing disease risk (e.g. Lyme borreliosis: Schmidt and Ostfeld 2001; *Batrachochytrium dendrobatidis*: Searle et al. 2011), others emphasize the role of species identity through relative host competence (e.g. amphibians affected by *Ribeiroia ondatrae*: Johnson et al. 2008) or abundance (e.g. small mammals reservoirs of *Borrelia*: Levi et al. 2012).

Community structure may play an important role in the evolution of generalist or specialist pahogens. Pathogens such as *Borrelia* may exhibit multiple niche polymorphisms, that is, different strains exhibit different fitness values (*R*_0_) in alternate hosts (Kurtenbach et al. 2006). As a result, frequency-dependent selection can favour different strains of *Borrelia* in variable vector-host systems. In species-rich systems, theory predicts that strains of pathogens will specialize (Woolhouse et al. 2001). Alternatively, generalist strains can be maintained through balancing selection or migration (Kurtenbach et al. 2006). Generalist strains are associated with depauperate faunal assemblages that may affect the rate at which pathogens can spread (Hanincová et al. 2006), pathogen virulence and the probability of infecting novel hosts (Woolhouse et al. 2001). A theoretical model by Roche et al. (2012) suggests that the mean susceptibility in the host community, which depends on the composition and relative abundance of species, has a positive effect on disease prevalence. These authors also show that higher species diversity increases the number of infected hosts, but decreases their proportion in the community. Biodiversity loss as a response to climate warming may equally occur in parasites and disease vectors themselves, which would result in a decrease in disease prevalence (Rohr et al. 2011). Therefore, changes in host communities may lead to opposite outcomes in disease risk depending on ecological circumstances.

Host–pathogen relationships may also respond to environmental changes through local adaptation to novel environmental conditions, which is ultimately an alternative to extinction or range expansion (e.g. evolutionary rescue; Vander Wal et al. 2013b). As previously mentioned, a classic example of co-evolution in a host–pathogen system involves diseases in European rabbits introduced to Australia. An additional example of the interplay between host and pathogen includes chytrid fungus (*B. dendrobatidis*; Bd), which devastated anuran populations. Recent evidence suggests that during the geographic spread of the fungus its virulence has changed. Specifically, the lag between invasion of the fungus and resulting population decline has diminished markedly over time, indicating a change in virulence along the invading front (Phillips and Puschendorf 2013). Data from genome resequencing further suggest that fungus lineages are older, more diverse and exhibit more heterogeneous and dynamic genomic architecture than previously documented (Rosenblum et al. 2013). Furthermore, Rosenblum et al. (2013) found enrichment in gene families that may be related to pathogenicity and are under selection. Although the underlying causes of reduced virulence in Bd remain unknown (e.g. ecological factors, host or pathogen evolution), it is evident that a more detailed integration of the disease evolutionary history and adaptive potential of this disease could help predict and mitigate its impact.

## Synthesizing evolutionary applications

Applied evolution can be decomposed into four main categories: phenotypic and genetic variation, evolutionary history, selection and eco-evolutionary dynamics (Hendry et al. 2011; Lankau et al. 2011; Vander Wal et al. 2014). Detecting evolution in wild host populations involves techniques and tools founded on these four principles. Those most frequently employed tools rely heavily on within and between species genetic variation that has arisen through evolutionary processes (e.g. selection and drift). In the following section, we highlight a number of examples where these tools and principles have been employed to understand better the host–pathogen dynamics (e.g. historical occurrence or spatial patterns of spread). Often inferences gained from these techniques are a cost-effective means to develop science-based management actions.

Approaches that rely on variation in molecular markers are useful to decompose the evolutionary and ecological linkages of the epidemiological triangle (Fig. 2). The use of molecular markers for species identification can also bring key insights in epidemiology by facilitating the study of prevalence patterns of pathogens and parasites in wildlife (Baillie et al. 2012). When outbreaks of emerging infectious diseases occur, pathology can often identify the cause of death, but may not be able to identify the causative agent. Molecular markers can be used to identify cryptic species, when different species of pathogens or parasites are morphologically indistinguishable – especially as eggs or larvae stages. For instance, the mitochondrial cytochrome *b* gene in avian malaria parasites (genera *Plasmodium* and *Haemoproteus*) revealed that several lineages often co-exist in a single host and that there may be as many avian malaria lineages as there are bird species (~10 000), in sharp contrast with the approximately 175 species recognized morphologically at the time (Bensch et al. 2007; Harrigan et al. 2014).

Once pathogens are detected in host populations, it is critical to evaluate their risk of spreading within the population and among neighbouring populations (Biek and Real 2010). Landscape genetics approaches have been widely applied to assess how landscape characteristics affect gene flow (Storfer et al. 2010) and have recently been used to examine questions related to infectious diseases and epidemiology (Real and Biek 2007). For instance, studies have combined temporal and spatial genetic data, often using microsatellite markers, with landscape modelling to examine routes for transmissions, as well as hosts and pathogen population structure. Results suggest that barriers and fragmented landscapes can restrict or facilitate disease spread or parasite invasion (Blanchong et al. 2008; Su et al. 2008; Vander Wal et al. 2012, 2013a) and modulate potential outbreaks by shaping hosts population structure (Cullingham et al. 2009; Rioux-Paquette et al. 2014). Such partitioning of variation may help to define the scale at which we should study demographic and evolutionary processes.

In some instances, managers may want to influence population connectivity to mitigate the possibility of pathogen spread (Hess 1994). Such an approach requires a fine-scale understanding of host movement. Using landscape genetics, one can evaluate the most parsimonious sets of factors affecting gene flow relative to one another to establish their effect on dispersal rates and functional connectivity (Taylor et al. 1993). To do so, causal modelling is often used to test different alternative hypotheses of landscape connectivity (Cushman et al. 2006) and least-cost paths are integrated in these models using habitat-specific costs to choose the most parsimonious model to explain the genetic distances documented among clusters or individuals. Isolation-by-resistance (IBR) models (McRae 2006), based on circuit theory, are also used to assign a resistance values to each element of the landscape and then compute resistance distances between pairs of individuals on the surface (see Dudaniec et al. 2013). Importantly, these analyses allow the construction of maps allowing to visualize for a given study area the most likely routes of dispersal of hosts and thus the potential spread of disease (Rioux-Paquette et al. 2014).

Phylogenetics, the reconstruction of evolutionary histories and relationships among taxa on the basis of genetic variation, has provided several useful applications to the study of infectious diseases (Brooks and Hoberg 2006; Hall and Barlow 2006). For example, pathogen phylogenetics revealed the role of human-induced movement in the spread of amphibian ranavirus in North American salamanders, possibly via fishing baits (Jancovich et al. 2004). Such information can lead to the prohibition of transporting live individuals among jurisdictions for bait. Phylogenetic techniques also identified the parallel emergence of hypervirulent chytrid fungus lineages from a single clade on five continents (Farrer et al. 2011) and documented the role of farmed salmon in the transmission of piscine reovirus in wild populations (Garseth et al. 2013). Phylogenetic analysis remains central in determining the origins of epizootics crossing from wildlife or livestock to humans (e.g. HIV, Keele 2006; West Nile virus, Lanciotti et al. 1999). Robust phylogenies for pathogens allow scientists to test hypotheses about cross-species transmission (Faria et al. 2013) and changes in vector preference, and about past adaptations of different lineages. Ultimately, phylogenetic analyses provide information on the likelihood of similar events occurring in the future (Rich and Xu 2011). Phylogenetics is a useful tool to help managers predict the probability of cross-species transmission; however, it is likely more challenging to then predict the strength of response a naïve species will exhibit when exposed to a novel pathogen. In such a scenario, epidemiological data from closely related species of host and pathogen may be the only information available for risk assessment.

Coevolution of host–pathogen dynamics in applied wildlife scenarios is less well understood, if no less important. A key aspect of molecular epidemiology is the study of coevolution, where speciation in a host leads to speciation in the pathogens, parasites or symbionts associated with it (Brooks 1979). Codivergence should lead to congruence between the phylogenetic tree of hosts and pathogens/parasites, and hence, cophylogenetics approaches allow tests of hypotheses regarding pathogen/host coevolution and preferential host switching (Charleston and Robertson 2002; Cuthill and Charleston 2013). Phylogenetic trees can also be combined with ecological, spatial or epidemiological data to calculate indices of host specificity (Poulin et al. 2011) and to infer transmission trees of the disease in host populations (Morelli et al. 2012).

An interesting recent development in molecular epidemiology is the emergence of the field of phylodynamics (Grenfell et al. 2004; Pybus and Rambaut 2009). Several pathogens (most notably viruses, but also bacteria) are characterized by typically high evolutionary rates. Consequently, their evolutionary and ecological dynamics occur on similar timescales: genomic diversity and adaptation can emerge within a few days. Statistical models at the interface of phylogenetics and population genetics that incorporate notions such as coalescent theory and relaxed molecular clocks (e.g. BEAST software; Drummond and Rambaut 2007) can thus be applied on pathogen genetic or genomic data to jointly estimate evolutionary parameters such as the timing of emergence of a given lineage or the estimated population size of a pathogen. Because of the interaction of evolutionary and ecological processes in such organisms, these evolutionary trajectories also provide insights into infection and transmission dynamics. For instance, the topology of a pathogen phylogenetic tree is influenced by the contact structure within host populations (Leventhal et al. 2012). While most phylodynamic studies have been applied to global human diseases (but see Biek et al. 2007 with wildlife), we expect emerging wildlife diseases to be the focus of such work in upcoming years.

## Future directions and challenges

While great advances have been made in the field of landscape epidemiology and phylogenetic analyses both within and across species (Benavides et al. 2014), most documented patterns of genetic variation are obtained using neutral genetic markers, a rather indirect way of assessing host ‘immune condition’ and the selective pressures affecting host-parasites systems. Recent reviews (Manel and Holderegger 2013) highlight a need to improve our understanding of the processes underlying patterns of genetic diversity by using a more predictive approach and assessing adaptive genetic variation. A first way of achieving this is by conducting more studies to quantify the extent of additive genetic variance underlying immunity-related traits and/or nonimmunological mechanisms such as behaviour that contribute to host defence (*sensu* Parker et al. 2011). For example Maze-Guilmo et al. (2014) quantify the underlying variance in immunological resistance and tolerance transmitted between generations. Transmitted variance includes additive genetic variance, epigenetic and social transmission (Danchin et al. 2011). Although these authors find that tolerance and resistance do not co-vary, they are both equally heritable. Indeed, despite several quantitative genetics studies in the wild, evidence of a genetic basis for immunity-related traits remains equivocal and mainly restricted to potential hosts (Graham et al. 2010). To achieve more realistic assessment of hosts and parasites evolutionary potential, we also need to assess the stability of the additive variance underlying these traits across ecological contexts (Charmantier and Garant 2005).

Another way of improving our understanding of the processes underlying the patterns of genetic diversity is through the development and application of landscape genomics to identify loci related to immunity and assess the strength of selection acting on them. The availability of large single nucleotide polymorphism (SNP) data sets has led to techniques for genomewide association studies (Segura et al. 2012) and candidate gene approach (Brown et al. 2012), where associations between SNPs and traits can point to sections of genomes/genes under selection or involved in resistance to pathogens. Even more to the point would be to associate immune traits of interests with specific genes or genomic features and to assess causality of the relationships in experimental conditions (Pavey et al. 2012).

Finally, a more comprehensive integration of hosts and parasites life histories (Barrett et al. 2008) and the incorporation of more dynamic information during successive stages of invasion (Meentemeyer et al. 2012) into theoretical and empirical studies of disease variation would improve our understanding of patterns of selection and evolution. Previous research on coevolutionary interactions showed that parasite local adaptation to hosts is typically strongest when gene flow rates are higher in the parasite than in the host and when gene flow in the host is low overall (Hoeksema and Forde 2008; for review see Poulin and Forbes 2012). Alternately, the effect of gene flow for host adaptation to parasites can be positive, negative or nonexistent (Garant et al. 2007; Hoeksema and Forde 2008). Recent studies suggest that temporal and spatial scales of divergence might differ in such systems (Torres-Pérez et al. 2011), reinforcing the need for a better integration of information on host and pathogen life history, as well as their dynamics.

Evolutionary responses to disease management have been described as a ‘black box’ (Joseph et al. 2013). These authors briefly examine the two main concerns: evolution of vaccine resistance and implications of selective culling. Wildlife managers have a limited number of tools to deal with outbreaks of disease (Wobeser 2002). When outbreaks might have negative implications for population health or pose risks to a region’s economy, managers are compelled to act. For some diseases, such as rabies, large-scale vaccination programs are commonplace (Rees et al. 2011). However, pathogens have been known to evolve vaccine resistance (Gandon et al. 2003), questioning the long-term efficacy of such approaches. Whereas the response to disease in an agricultural setting is whole-herd depopulation, similar extirpation of species in the wild is typically infeasible, arguably unethical (Crozier and Schulte-Hostedde 2014) and likely has detrimental cascading ecological implications. An alternative is ‘test and cull’ programs (e.g. bovine tuberculosis, Brook et al. 2013). Here, only animals that test positive or are suspected of being infected with disease are removed from the population. However, selective culling acts as a selective pressure that may result in increased virulence and disease prevalence (Bolzoni and De Leo 2013).

Unfortunately, exotic wildlife diseases often involve high rates of host mortality, sometimes because of complex interactions with ecological variables such as new and favourable abiotic conditions and new species of carriers affecting prevalence and transmission (Puechmaille et al. 2011). Consequently, management of wildlife disease involves difficult challenges: prevention may require strict and costly surveillance and limiting the spread of established diseases will require consideration of possible evolutionary changes of the disease organism, alternative host species and complex interactions with a novel environment. Many diseases that can be relatively easily controlled in captive or domestic animals are very difficult to control in the wild (Krkošek et al. 2006). Yet, the risks of new wildlife diseases cannot be ignored, as they have potentially devastating consequences for wild and domestic species with substantial economic value (Langholz and Jay-Russell 2013), can have a detrimental effect on biodiversity and ecosystem services (Puechmaille et al. 2011) and may in some cases lead to new zoonoses with serious implications for human health (Daszak et al. 2000; Woolhouse et al. 2005).

Of particular relevance to wildlife disease management is farming of species taxonomically similar to wild ones which can share pathogens. Transmission between domesticated species and sympatric wildlife underpins the emergence of a range of wildlife diseases. Examples where the main reservoir is a domestic species are numerous, ranging from pneumonia transmitted from domestic to bighorn sheep (*Ovis canadensis*) (Cassirer et al. 2013), cheratoconjunctivitis and brucellosis transmitted from domestic sheep to wild ungulates in Europe (Giacometti et al. 1998), and salmon lice transmitted from farmed to wild salmon (Krkošek et al. 2006; Miller et al. 2014). These diseases can be controlled in domestic stock through vaccines or antibiotics but are nearly impossible to control in wild animals where they can cause devastating mortality. The reverse situation, where disease is transmitted from wild reservoirs to domestic animals, can also occur, although it is in many cases controversial as politicians and other vested interests find it simpler to blame wildlife species rather than addressing more complex causes of epizootics that require changes in farming practices. Examples include brucellosis and tuberculosis in wild bison (*Bison bison*), elk (*Cervus canadensis*) and domestic cattle in North America (Wobeser 2009) and tuberculosis in badgers (*Meles meles*) and livestock in Britain (Donnelly et al. 2006). Worldwide, rabies is maintained by wild hosts (Baer 1991). In the specific case of raccoons in North America, rabies provides a very rare example of a wildlife disease that can be effectively controlled with a massive vaccination programme (Rees et al. 2011), because of the availability of both an effective vaccine and an effective bait to deliver it (Boyer et al. 2011). Vaccines, however, require uncommon circumstances to be widely applicable to wildlife disease (Cross et al. 2013).

Many wildlife diseases are a direct health concern to humans. For example, rabies, malaria and plague have caused human mortality for centuries and have wild reservoirs (Baer 1991). Recently, however, human activities have facilitated the spread of serious disease that involve a wildlife reservoir. Examples include the introduction of West Nile virus in North America (Kilpatrick et al. 2006), epidemics of SARS (originating from a civet, *Paguma larvata*; Bell et al. 2004) and ‘bird flu’ (Chen et al. 2005). In other cases, human diseases are increasing their range as global warming allows the northward shift of their wildlife host, for example Lyme disease (Ogden et al. 2009; and see above). Management of these emergent diseases is therefore essential. Efficient control strategies require a multidisciplinary approach that incorporates evolutionary principles (Karesh et al. 2012).

## Concluding remarks

It is increasingly being accepted that, due to rapid evolution, ecological and evolutionary properties in a system change in tandem (Smallegange and Coulson 2013). Indeed, this has been demonstrated for a number of ecological processes across diverse systems (Pelletier et al. 2007; Coulson et al. 2011). These codynamics on a converged timescale have resulted in calls for evolutionarily enlightened management (Ashley et al. 2003). Nowhere are these codynamics more prevalent than in host–pathogen systems. As a result, calls to better integrate evolutionary principles – such as evolutionary history, variation, selection and eco-evolutionary dynamics – into the management of infectious disease are becoming more frequent (Grenfell et al. 2004; Karesh et al. 2012; Joseph et al. 2013). Nevertheless, these principles remain infrequently employed or factored into decision-making. Possible exceptions include the use of evolved patterns of genetic diversity across landscapes to understand host movements as a proxy for landscape-scale transmission risk (Blanchong et al. 2008; Lee et al. 2012). Similarly, some exemplar studies have integrated the principles of pathogens as possible agents of selection (May and Anderson 1983; Ujvari et al. 2012; Lagagneux et al. 2014). However, many opportunities remain to employ the four principles of applied evolution to the study and management of wildlife disease. For example, understanding how landscape change can act as a selective pressure on host-pathogen dynamics; or how pathogens can induce adaptation in life-history traits, behaviour, resistance or tolerance and their consequent implications for host population dynamics. Furthermore, the down-stream implications of disease management practices (e.g. vaccination or culling), also merit careful reflection. Ultimately, an evolutionarily enlightened perspective on wildlife disease, such as the eco-evo epidemiological triangle, should better inform management and conservation practices.
